# Acute Symptoms after a Community Hydrogen Fluoride Spill

**DOI:** 10.1186/2052-4374-25-17

**Published:** 2013-09-19

**Authors:** Joo-Yong Na, Kuck-Hyun Woo, Seong-Yong Yoon, Seong-Yong Cho, In-Ung Song, Joo-An Kim, Jin-Seok Kim

**Affiliations:** 1 Department of Occupational and Environmental Medicine, Soonchunhyang University Gumi Hospital, 179, Gongdan 1-dong, Gumi-si, Gyeongbuk 730-706, Korea

**Keywords:** Hydrofluoric acid, Chemical hazard release, Signs and symptoms

## Abstract

**Objectives:**

This study was conducted to describe the demographic characteristics, and clinical signs and symptoms of patients who visited a general hospital because of the release of chemically hazardous hydrogen fluoride that occurred on September 27, 2012 in Gumi City, Korea.

**Methods:**

The medical records at 1 general hospital 9 km from the accident site were reviewed using a standardized survey format. There were 1,890 non-hospitalized and 12 hospitalized patients exposed to hydrogen fluoride between September 27 and October 13 2012.

**Results:**

Among the 12 hospitalized patients, 11 were discharged within 1 week and the other was hospitalized for 10 days. The chief complaints were respiratory symptoms such as hemoptysis and shortness of breath, gastrointestinal symptoms, neurologic symptoms, sore throat, and lip burn.

The number of non-hospitalized patients exhibited a bimodal distribution, peaking on the first and twelfth days after the accident. Their chief complaints were sore throat (24.1%), headache (19.1%), cough (13.1%), and eye irritation (9.2%); some patients were asymptomatic (6.2%). Patients who visited the hospital within 3 days (early patients) of the spill more often had shortness of breath (27.0%) and nausea (6.3%) as the chief complaints than patients who visited after 3 days (late patients) (3.5% and 2.6%, respectively). However, cough and rhinorrhea were more common in the late patients (14.0% and 3.3%, respectively) than in the early patients (5.0% and 0.0%, respectively). Patients who were closer to the accident site more often had shortness of breath and sputum as the chief complaints than patients who were farther away. The mean serum calcium concentration was 9.37 mg/dL (range: 8.4–11.0 mg/dL); none of the patients had a decreased serum calcium level. Among 48 pulmonary function test results, 4 showed decreased lung function. None of the patients had abnormal urine fluoride levels on the eighth day after exposure.

**Conclusions:**

Patients hospitalized due to chemical hazard release of hydrogen fluoride had acute respiratory, gastrointestinal, and neurologic health problems. Non-hospitalized patients have acute symptoms mainly related to upper respiratory irritation.

## Introduction

Hydrogen fluoride is a highly hydrophilic and corrosive irritant that may cause burns to the skin and eyes and may damage the upper respiratory tract and lungs when inhaled. Hydrogen fluoride and its aqueous solution, hydrofluoric acid, can infiltrate the body via all exposure routes. It is less acidic than inorganic acids such as hydrogen chloride and hydrogen sulfide. However, its high permeability can be fatal and cause deep tissue damage after a certain period of time from the initial contact with skin [1-6]. The most representative chronic toxicities of hydrogen fluoride are osteofluorosis and dental fluorosis, which involve the deposition of fluoride in the bones and teeth in people constantly consuming fluoride-contaminated drinking water or in workers performing aluminum smelting who are chronically exposed to hydrogen fluoride [7,8]. Hydrogen fluoride has been used in glasswork since the 17th century and is currently used in the manufacturing of high-octane gasoline for aviation fuel, metal plating, and high-temperature surface processing as well as in the production of plastics and refrigerants. In particular, hydrogen fluoride is capable of etching glass and metals, which prompts its widespread use in the semiconductor industry and in the manufacturing of electronic displays [1,9].

On September 27, 2012 at 3:40 pm, a hydrogen fluoride spill occurred in a hydrogen fluoride manufacturing plant located in Gumi City while 100% hydrogen fluoride (anhydrous hydrofluoric acid) was being transferred from a hydrogen fluoride tanker truck to a storage tank. An estimated 8–12 tons of hydrogen fluoride was leaked over approximately 8 h until the tank spill was completely stopped. This accident killed 5 workers on site and hydrogen fluoride spread through the air, damaging the health of local residents and industrial workers as well as crops and other facilities [10].

**Figure 1:**
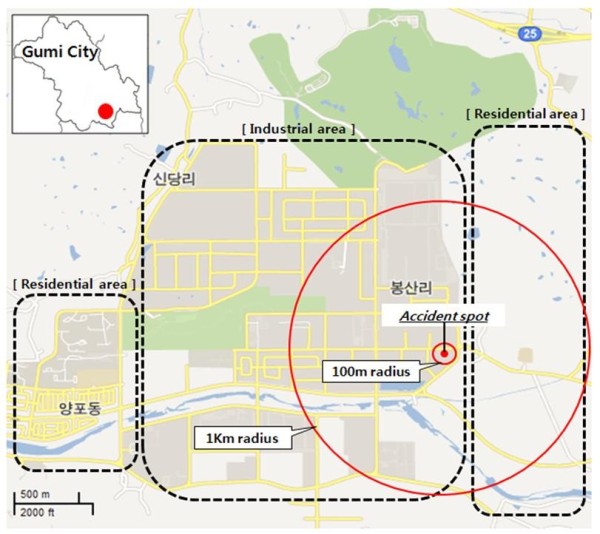
**Map of the Gumi industrial complex and the surrounding residential area.** The hydrogen fluoride spill area is designated by the red circle.

The accident site was located at the fourth complex of the Gumi National Industrial Complex; local residents lived within a 2 km radius (Figure 1). According to the Gumi municipal accident report, the estimated time of the accident was 3:40 pm. The first evacuation notice was issued to the residents living within a 1.5 km radius around 4:50 pm, followed by an evacuation order for all residents living within a 1.3 km radius at 8:20 pm. According to the report of the Gumi Municipal Government [10], the atmospheric hydrogen fluoride concentration at the accident site was first measured 8 h after the accident and was 1 ppm. However, the second measurement, which was conducted at the accident site 5 h later, did not detect hydrogen fluoride in the atmosphere or nearby residential areas. The results of the initial water and soil quality standard tests conducted on October 1 and 2 were both below the thresholds for concern (1.5 mg/L for water quality [11] and 400 mg/kg for soil quality [12]); subsequent tests also revealed the levels to be below the threshold values [13].

This study described and analyzed the demographic and clinical characteristics of patients who were treated at a general hospital for symptoms related to hydrogen fluoride exposure after the accident. In addition, we present the treatment experience of the outpatient clinic of the Department of Occupational and Environmental Medicine.

## Materials and methods

A total of 6,982 people were treated at a private medical institution for physical symptoms caused by the hydrogen fluoride spill from September 27 to October 21, 2012, and an additional 5,261 people visited the temporary on-site clinic provided by the Gumi Municipal Government; thus, a total of 12,243 people were treated for fluoride-related symptoms [10]. Among the 3 general hospitals near the accident site, this study analyzed patients treated for hydrogen fluoride exposure at a university hospital located approximately 9 km from the accident site. A total of 1,952 outpatients visited the university hospital over 17 days from the start of the accident (from September 27 to October 13, 2012) because of symptoms related to hydrogen fluoride exposure, including 3 people who died. There were 10 hospitalized patients during the same period, and an additional 2 were hospitalized after October 14, resulting in a total of 12 hospitalized patients. Medical records and questionnaires completed at the time of outpatient treatment of these patients were reviewed by 7 clinicians at the Department of Occupational and Environmental Medicine using the same survey method. A total of 1,952 outpatients were surveyed; 1,890(96.8%) were included in the final analysis after excluding the 62 patients for whom medical records were unavailable.

A questionnaire survey was conducted on the exposure characteristics and symptoms. But, the survey was not conducted at the emergency clinic, because the questionnaire could not be prepared. Outpatients who attended 6 days after the accident occurred were surveyed through a self-completed questionnaire. When completing the survey, prior consent was obtained using a consent form for the collection and use of personal information. The surveyed content included basic demographic information (e.g., occupation and residence), exposure characteristics after the spill (e.g., distance from the accident site and location other than indoors), main symptoms, accompanying symptoms, medical history, physical examination findings, prescriptions, and second visits.

During outpatient visits at the Department of Occupational and Environmental Medicine, regular blood tests, liver function index tests, electrolyte tests including serum calcium, chest radiographs, pulmonary function tests, and urine fluoride concentration tests were performed with patient consent. No tests were performed on patients who were asymptomatic, showed mild symptoms, or did not provide consent. Many patients who initially visited the hospital through the emergency room received treatment simultaneously and required immediate calcium gluconate inhalation therapy and eye wash treatment. Therefore, information regarding exposure, symptoms, and clinical test results could not be identified through the medical records of the majority of the patients who initially visited the emergency room.

Urine fluoride was measured as a biological index of exposure to quantify individual exposure. Immediately after the spill, there were no institutions in Korea that could immediately perform fluoride analysis. On October 5 (the eighth day after the spill), a urine fluoride analysis device was ordered. Sixty-six patients suspected of having been exposed to relatively high concentrations of hydrogen fluoride among the 217 patients who visited on that day were selected; these patients included 46 workers who normally worked at an office within 100 m of the accident site even after the spill and 20 patients who were arbitrarily selected by clinicians because of their severe symptoms. Urine samples were stored in a freezer at −20°C and analyzed using a fluoride-ion specific electrode analyzer (Orion Star A214, Thermo Fisher Scientific Inc., Japan) when analysis was possible after 2 weeks.

With respect to the occupations, the patients were classified as local residents, workers, firefighters, police, public officials, and others. Local residents were further classified into adjacent residents and other residents. Adjacent residents included those from the 2 villages close to and downwind of the accident site that suffered direct damage to crops and facilities. The rest were classified as other residents. Depending on the time of the hospital visit, those who visited within 3 and after 4 days of the accident were classified as early and late patients, respectively. In addition, the distance to the accident site was classified as within 100 m, from 100 m to 1 km, and over 1 km.

Data were analyzed using SPSS version 14 (SPSS, Inc., Chicago, IL, USA). The χ2 test and t-test were used for simple analyses, while χ2 trend analysis was used to analyze differences in symptoms and complaints with respect to the distance from the accident site. Analysis of variance (ANOVA) and analysis of covariance (ANCOVA) adjusted for age and sex were used to compare clinical parameters.

## Results

A total of 12 patients were hospitalized owing to hydrogen fluoride exposure. Among them were 5 residents in the nearby area, 4 workers, 2 other residents, and 1 public officer. Eleven patients showed improvement after appropriate treatment and were discharged within 1 week, but 1 patient who was admitted on October 16 was hospitalized for 10 days. The chief complaints of the hospitalized patients were respiratory symptoms such as hemoptysis and dyspnea; gastrointestinal symptoms including nausea and indigestion; neurologic symptoms such as headache and numbness; sore throat; and lip pain. Three patients hospitalized due to hemoptysis presented with redness and edema in the bronchial mucosa and rebleeding during washing on bronchoscopy. However, they were discharged after several days of conservative treatment. The 2 patients who complained of gastrointestinal symptoms presented with ulcers and chronic atrophic gastritis, respectively, during endoscopic examination but were discharged when they showed improvement after medication. The patient who was hospitalized at the otorhinolaryngology department because of findings of acute throat injection was discharged after medication. No specific abnormalities were found on physical examinations or by clinical tests in the patients hospitalized in the respiratory and neurology departments for symptoms such as shortness of breath, headache, and numbness in the hands and feet; these patients were discharged after receiving conservative treatment for their symptoms (Table 1).

**Table 1:** Details of the 12 hospitalized patients

**Age**	**Gender**	**Department**	**Date of admission**	**Date of discharge**	**Chief complaints**	**Diagnosis**	**Exposure characteristics**	**Clinical findings**
30	M	IP^†^	9/28	9/29	Dyspnea	Gas inhalation	Worker	No abnormal findings
51	F	IP	10/3	10/10	Hemoptysis	Bronchitis	Adjacent resident	Bronchoscopy ; hyperemic, both bronchi edematous
52	M	IP	10/3	10/8	Blood-tinged sputum	Bronchitis	Governmental officials	Bronchoscopy ; both bronchi hyperemic
44	F	IP	10/9	10/12	Dyspnea	Gas inhalation	Worker	No abnormal findings
52	F	IP	10/16	10/25	Dyspnea	Toxic effect of HF^*^	Adjacent resident	Anxiety
38	M	IP	10/24	10/26	Hemoptysis	Bronchitis	Worker	Bronchoscopy ; both bronchi hyperemic
44	F	IG^‡^	10/6	10/10	Nausea	Gastric ulcer	Adjacent resident	GIF^**++**^; gastric ulcer
68	F	IG	10/9	10/12	Nausea	Gas inhalation	Adjacent resident	GIF; chronic atropic gastritis
25	F	ENT^§^	10/6	10/11	Sore throat	Acute pharyngotonsillitis	Worker	Pharyngeal injection
57	F	NR^**‖**^	10/4	10/8	Numbness	Numbness	Other resident (Intake of contaminated crops)	No abnormal findings
79	F	NR	10/9	10/12	Headache	Chronic tension headache	Adjacent resident	No abnormal findings
71	F	PS^**^	10/8	10/8	Lip pain	Chemical burn on lower lip	Other Resident (intake of contaminated crops)	Swelling, erythema on lower lip

^*^Hydrogen fluoride, ^†^Internal medicine, pulmonology, ^‡^Internal medicine, gastroenterology, ^§^ENT; Otorhinolaryngology, ^**‖**^Neurology, ^**^Plastric surgery, ^**++**^Gastro-intestinal fiberscopy.

**Figure 2:**
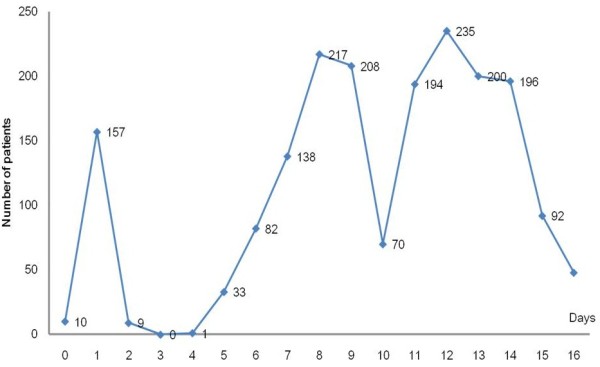
Distribution of numbers of non-hospitalized patients by day.

Regarding the distribution of time of hospital visits, 10 patients visited on the day of the accident (September 27) through the emergency room, followed by 157 and 9 patients on the first and second days after the accident, respectively. No patients visited the hospital on the third day after the accident (September 30), which was the Chuseok Holiday. From the fifth day after the accident, the number of visiting patients increased to 33, reaching 217 on the eighth day, decreasing to 70 on the tenth day, and then decreasing again after reaching a peak of 235 patients on the twelfth day (Figure 2).

Regarding the age distribution of the outpatients, the largest proportion of patients were in their 30 s (30.4%) followed by patients in their 40 s (21.2%), 20 s (17.9%), and those aged <9 years (5.8%). Men accounted for 52.8% of all patients. The departments that provided the initial treatment were occupational and environmental medicine (80.8%), pediatrics (7.2%), internal medicine (6.9%), emergency medicine (2.9%), ophthalmology (1.1%), and otorhinolaryngology (0.6%). Among the patients exposed to hydrogen fluoride, the largest proportion were residents (42.7%), with 11.2% and 31.5% of patients representing nearby and other residents, respectively; workers, police and public officials, and firefighters accounted for 39.9%, 3.6%, and 2.1% of the patients, respectively. At the time of the accident, 80.4% and 19.1% of the patients exposed to hydrogen fluoride were indoors and outdoors, respectively. A total of 176 (9.3%) patients visited the hospital within 3 days of the accident, while 1,714 (90.7%) visited 4 days after the accident, and these patients were classified as early and late patients, respectively. Regarding the distance from the accident site, 14.6%, 46.5%, and 38.9% of patients were within 100 m, 100 m to 1 km, and over 1 km, respectively, from the accident site (Table 2).

**Table 2:** General characteristics of non-hospitalized patients

**Characteristic**	**Number**	**%**
Age(years)		
0–9	109	5.8
10–19	80	4.2
20–29	338	17.9
30–39	575	30.4
40–49	400	21.2
50–59	253	13.4
≥60	135	7.1
Gender		
Male	997	52.8
Female	893	47.2
First visit to the outpatient department		
Occupational and environmental medicine	1528	80.8
Pediatrics	136	7.2
Internal medicine, Pulmonology	130	6.9
Emergency medicine	55	2.9
Ophthalmologic medicine	22	1.1
Otorhinolaryngology	11	0.6
Others	9	0.6
Exposure characteristics		
Adjacent residents	205	11.2
Other residents	575	31.5
Workers	728	39.9
Firefighters	38	2.1
Police and public officials	66	3.6
Others	211	11.6
Location at the time of the accident		
Indoor	922	80.4
Outdoor	219	19.1
Others	6	0.5
Time of first hospital visit		
Within 3 days	176	9.3
After 4 days	1714	90.7
Distance from the place of the accident spot		
Under 100 m	227	14.6
100 m-1 km	721	46.5
Over 1 km	603	38.9

The chief complaints reported by the patients were sore throat (24.1%), headache (19.1%), cough (13.2%), and eye irritation (9.2%); 6.2% of the patients were asymptomatic. The prevalence of shortness of breath was higher among early patients (27.0%) than among late patients (3.5%); nausea was also more prevalent in the early patients (6.3%) than the late patients (2.6%). In contrast, cough was more prevalent in the late patients (19.4%) than the early patients (5.0%). Moreover, there were more asymptomatic patients among the late patients (6.4%) than the early patients (3.8%) (p < 0.05) (Table 3).

**Table 3:** Chief complaints of early and late patients

**Chief complaint**	Early patients^*^		**Late patients**^**†**^		**Total**		**p-value**^**‡**^
	**N**	**%**	**N**	**%**	**N**	**%**	
Sore throat	37	23.3	394	24.2	431	24.1	0.797
Headache	25	15.7	316	19.4	341	19.1	0.260
Cough	8	5.0	228	14.0	236	13.2	0.001
Eye irritation	17	10.7	147	9.0	164	9.2	0.487
No symptom	6	3.8	105	6.4	111	6.2	0.183
Shortness of breath	43	27.0	57	3.5	100	5.6	0.000
Chest pain	3	1.9	63	3.9	66	3.7	0.206
Dizziness	2	1.3	54	3.3	56	3.1	0.155
Nausea	10	6.3	43	2.6	53	3.0	0.010
Rhinorrhea	0	0.0	53	3.3	53	3.0	0.021
Skin burning	2	1.3	36	2.2	38	2.1	0.427
Itching	0	0.0	32	2.0	32	1.8	0.075
Sputum	1	0.6	26	1.6	27	1.5	0.340
Nasal pain	1	0.6	14	0.9	15	0.8	0.761
Skin rash	0	0.0	7	0.4	7	0.4	0.408
Vomiting	0	0.0	2	0.1	2	0.1	0.658
Others	4	2.5	52	3.2	56	3.1	0.640
Total	159	100.0	1629	100.0	1788	100.0	

* Early patients: hospital visit within 3 days.

† Late patients: hospital visit 4 days or after.

‡ Chi-squared test.

Regarding individual symptom complaints other than chief complaints, the most common complaints were cough (43.3%), sore throat (42.6%), and headache (41.9%). Shortness of breath was observed in 30.7% of the early patients but in only 14.7% of the late patients. Cough (15.3% and 46.1% in the early and late patients, respectively), headache (26.7% and 43.5%), eye irritation (19.9% and 31.3%), dizziness (5.1% and 15.2%), and skin rash (0.6% and 3.8%) were more prevalent in the late patients (Table 4).

**Table 4:** Symptom complaints of early and late patients

	Early patients^*^		**Late patients**^**†**^		**Total**		**p-value**^**‡**^
	**N**	**%**	**N**	**%**	**N**	**%**	
Cough	27	15.3	791	46.1	818	43.3	0.000
Sore throat	64	36.4	741	43.2	805	42.6	0.079
Headache	47	26.7	745	43.5	792	41.9	0.000
Eye irritation	35	19.9	536	31.3	571	30.2	0.002
Nausea	25	14.2	327	19.1	352	18.6	0.114
Shortness of breath	54	30.7	252	14.7	306	16.2	0.000
Dizziness	9	5.1	260	15.2	269	14.2	0.000
Chest pain	5	2.8	102	6.0	107	5.7	0.089
Skin burning	5	2.8	77	4.5	82	4.3	0.306
Skin rash	1	0.6	65	3.8	66	3.5	0.027
Vomiting	2	1.1	45	2.6	47	2.5	0.227
Total	176	100.0	1714	100.0	1890	100.0	

* Early patients: hospital visit within 3 days.

† Late patients: hospital visit 4 days or after.

‡ Chi-squared test.

The patients’ characteristics were compared with respect to their distance from the accident site (i.e., within 100 m, 100 m to 1 km, and over 1 km). There were more men near the accident site: 18.3% of men were within 100 m compared to 10.6% of women. Regarding age, 63.3% of patients aged <19 years were located over 1 km away, while 11.8% and 21.2% of those aged 20–39 and 40–59 years were located within 100 m, respectively. Regarding those aged 60 years and over, 72.8% were located from 100 m to 1 km from the accident site. Regarding exposure characteristics, nearby residents accounted for a substantial number of patients, as 79.7% were located from 100 m to 1 km from the accident site, while 73.7% of the other residents were located over 1 km away. Regarding workers, 14.8% and 58.5% were within 100 m and from 100 m to 1 km, respectively. Among the early and late patients, 52.9% and 12.8% were within 100 m, respectively.

Physical examination findings were analyzed with respect to the distance from the accident site. Abnormal pulmonary sound was more common in patients closer to the site: 7.4%, 2.5%, and 1.0% were within 100 m, from 100 m to 1 km, and over 1 km away, respectively, from the site. Skin abnormalities were also common in the patients closer to the site: 9.7%, 6.7%, and 3.5% were within 100 m, from 100 m to 1 km, and over 1 km away, respectively, from the site. Among the 48 patients administered the pulmonary function test, 4 exhibited abnormal findings; however, there was no significant association with exposure distance (Table 5).

**Table 5:** Patient characteristics with respect to distance from the accident site

	**Under 100 m**		**100m-1 km**		**Over 1 km**		p-value*
	**N**	**%**	**N**	**%**	**N**	**%**	
Sex							0.000
Male	149	65.6	418	58.0	249	41.3	
Female	78	34.4	303	42.0	354	58.7	
Age							0.000
Under 19	7	3.1	22	3.0	50	8.3	
20~39	96	42.3	351	43.7	370	61.3	
40~59	117	51.5	273	37.9	162	26.9	
Over 60	7	3.1	75	10.4	21	3.5	
Exposure characteristics							0.000
Adjacent residents	12	5.4	126	17.6	20	3.3	
Other residents	12	5.4	103	14.4	323	53.7	
Workers	103	46.6	408	56.9	187	31.1	
Firefighters	19	8.6	1	0.1	3	0.5	
Public officials	9	4.1	8	1.1	5	0.8	
Others	66	29.9	71	9.9	64	10.6	
Time of hospital visit							0.000
Within 3 days	37	16.3	25	3.5	8	1.3	
4 days or after	190	83.7	696	96.5	595	98.7	
Abnormal physical findings							
Throat	72/136	52.9	273/466	58.6	204/400	51.0	0.240
Nose	10/80	12.5	25/248	10.1	16/223	7.2	0.126
Eye	12/82	14.6	35/250	14.0	25/242	10.3	0.202
Lung	7/95	7.4	8/326	2.5	3/303	1.0	0.001
Heart	0/92	0.0	0/294	0.0	0/292	0.0	-
Skin	7/72	9.7	15/225	6.7	8/229	3.5	0.002
Abnormal PFT							
FVC%^†^	2/14	14.3	1/27	3.7	1/7	14.3	0.739
FEV1/FVC%^‡^	0/14	0.0	2/27	7.4	0/7	0.0	0.747

^*^ Chi-squared test for trend.

^†^ Decreased FVC < 80%.

^‡^ Decreased FVC/FEV1 ratio <70%.

Upon analyzing the chief complaints with respect to distance, shortness of breath was found in 8.0%, 4.2%, and 3.4% of patients within 100 m, from 100 m to 1 km, and over 1 km away, respectively, from the site. In contrast, no symptoms and rhinorrhea were more common with increasing distance from the accident site. No symptoms were observed in 1.8%, 3.6%, and 6.1% of patients within 100 m, from 100 m to 1 km, and over 1 km away, respectively, from the site. Meanwhile, rhinorrhea was observed in 0.9%, 1.9%, and 4.7% of patients within 100 m, from 100 m to 1 km, and over 1 km away, respectively, from the site (Table 6).

**Table 6:** Chief complaints with respect to distance from the accident site

	**Under 100 m**		**100 m-1 km**		**Over 1 km**		p-value^*^
	**N**	**%**	**N**	**%**	**N**	**%**	
Sore throat	51	22.7	195	28.2	131	23.5	0.660
Headache	46	20.4	142	20.5	106	19.0	0.549
Cough	27	12.0	87	12.6	82	14.7	0.234
Eye irritation	21	9.3	58	8.4	47	8.4	0.742
Shortness of breath	18	8.0	29	4.2	19	3.4	0.012
Chest pain	11	4.9	33	4.8	18	3.2	0.190
Dizziness	9	4.0	27	3.9	17	3.0	0.423
Nausea	3	1.3	26	3.8	14	2.5	0.784
Rhinorrhea	2	0.9	13	1.9	26	4.7	0.001
Skin burning	5	2.2	13	1.9	16	2.9	0.405
Itching	5	2.2	13	1.9	9	1.6	0.557
Sputum	8	3.6	7	1.0	10	1.8	0.289
Nasal pain	2	0.9	3	0.4	9	1.6	0.137
Skin rash	1	0.4	4	0.6	0	0.0	0.169
Vomiting	0	0.0	1	0.1	0	0.0	0.744
Others	12	5.3	16	2.3	20	3.6	0.548
No symptom	4	1.8	25	3.6	34	6.1	0.003
Total	225	100.0	692	100.0	558	100.0	

^*^ Chi-squared test for trend.

Regarding the results of the blood tests and clinical chemistry tests, RBC, hematocrit, phosphorus, potassium, r-GTP, ALP, glucose, blood urea nitrogen, creatinine, and uric acid differed significantly according to the distance from the accident site. However, after adjusting for age and sex, the differences were only significant for hematocrit, potassium, and glucose. Blood hematocrit values increased with decreasing distance, with the values being 44.1 ± 4.44%, 43.5 ± 4.25%, and 41.9 ± 4.15% in patients within 100 m, from 100 m to 1 km, and over 1 km away, respectively. Potassium levels also increased with decreasing distance, with the levels being 4.13 ± 0.34, 4.07 ± 2.03, and 4.04 ± 0.28 mmol/L in patients within 100 m, from 100 m to 1 km, and over 1 km away, respectively. Glucose levels also increased with decreasing distance, with the levels being 113.0 ± 48.5, 105.5 ± 32.9, and 100.1 ± 20.9 mg/dL in patients within 100 m, from 100 m to 1 km, and over 1 km away, respectively (p < 0.05) (Table 7).

**Table 7:** Laboratory results with respect to distance from the accident site

	**Under 100 m**			**100 m-1 km**			**Over 1 km**			**Crude p-value**	Adjusted p-value*
	**N**	**Mean ± SD**		**N**	**Mean ± SD**		**N**	**Mean ± SD**			
Complete blood cell count											
WBC (×10^3^/mm^3^)	166	6.97 ± 1.60		488	7.11 ± 1.97		471	6.99 ± 1.88		0.548	0.500
RBC (×10^6^/mm^3^)	166	4.88 ± 0.46		488	4.79 ± 0.48		471	4.70 ± 0.46		0.000	0.175
Hemoglobin (g/dL)	166	14.8 ± 1.73		488	14.7 ± 1.67		471	14.0 ± 1.65		0.000	0.172
Hematocrit (%)	166	44.1 ± 4.44		488	43.5 ± 4.25		471	41.9 ± 4.15		0.000	0.032
Platelet (×10^3^/mm^3^)	166	252.8 ± 55.9		488	246.8 ± 51.0		471	254.6 ± 50.7		0.057	0.101
Serum electrolyte											
Calcium (mg/dL)	172	9.39 ± 0.37		489	9.35 ± 0.36		471	9.32 ± 0.37		0.106	0.226
Phosphorus (mg/dL)	172	3.57 ± 0.58		489	3.59 ± 0.57		471	3.74 ± 0.63		0.000	0.438
Sodium (mmol/L)	165	141.9 ± 1.93		485	142.0 ± 2.06		471	141.7 ± 1.79		0.055	0.611
Potassium (mmol/L)	165	4.13 ± 0.34		485	4.07 ± 0.32		471	4.04 ± 0.28		0.006	0.044
Chloride (mmol/L)	164	102.9 ± 2.04		485	103.1 ± 2.03		471	103.1 ± 1.84		0.438	0.778
Serum chemistry											
AST^†^ (IU/L)	162	24.0 ± 10.8		476	25.3 ± 12.4		470	23.7 ± 13.5		0.161	0.310
ALT^‡^ (IU/L)	162	25.1 ± 16.6		476	26.9 ± 23.7		470	23.5 ± 25.1		0.087	0.466
r-GTP^§^ (IU/L)	155	36.8 ± 31.6		473	39.3 ± 52.3		466	27.5 ± 28.9		0.000	0.240
ALP^**‖**^ (IU/L)	158	180.6 ± 62.6		475	177.6 ± 51.2		467	195.8 ± 130.3		0.010	0.136
Glucose (mg/dL)	156	113.7 ± 48.5		461	105.5 ± 32.9		446	100.1 ± 20.9		0.000	0.038
Total cholesterol (mg/dL)	159	197.6 ± 36.0		475	194.7 ± 36.0		467	190.7 ± 35.5		0.065	0.816
Blood urea nitrogen (mg/dL)	161	13.9 ± 3.97		473	13.1 ± 3.89		468	12.7 ± 3.59		0.001	0.109
Creatinine (mg/dL)	162	0.88 ± 0.14		473	0.89 ± 0.18		469	0.81 ± 0.19		0.000	0.070
Uric acid (mg/dL)	152	5.62 ± 1.49		460	5.51 ± 1.47		444	5.12 ± 1.45		0.000	0.540

^*^Adjusted for age and sex, using ANCOVA test.

^†^Aspartate transaminase, ^‡^Alanine transaminase, ^§^Gamma glutamyl transpeptidase, ^**‖**^Alkaline phosphatase.

Calcium concentration was measured in 1,284 outpatients. The mean of the measured values was 9.37 mg/dL (range: 8.4–11.0 mg/dL). No patients had calcium levels below the threshold of 8.4 mg/dL. The mean urine fluoride level of the 66 samples collected on the eighth day after the accident was 0.39 ± 0.24 mg/L (range: 0.03–1.6 mg/L). All of the measurements were below 3 mg/g Cr, which is the pre-work exposure threshold provided by American Conference of Governmental Industrial Hygienists (ACGIH).

## Discussion

Hydrogen fluoride is a potent irritant that exists in a colorless gaseous or transparent liquid state. Pure hydrogen fluoride exists in a gaseous form at room temperatures over 19.5°C [14,15]. It is not explosive but generates heat when mixed with water; can react with metal, water, and steam; and can corrode silicon, glass, and concrete [9,16]. The Gumi spill involved 100% hydrogen fluoride. The fumes generated by the hydrogen fluoride combining with the moisture in the atmosphere were spread by the wind to adjacent areas, harming people, animals, and plants in the local community, as well as corroding buildings and cars due to their corrosive nature against metals and glass. Hydrogen fluoride which combines with the moisture in the mucous membranes of the human body can cause irritation first in the skin, eyes, and upper respiratory system including the nose and throat. Hydrogen fluoride gas is strongly hydrophilic and reactive in slightly acidic environments such as the body’s physiological acidity (pKa: 3.45); therefore, gas inhaled through the oral or nasal cavities is mostly absorbed into the upper respiratory tract. Inhaling large amounts of hydrogen fluoride gas can cause interstitial pneumonia and pulmonary edema, because the gas is absorbed by the lower respiratory tract as well [17,18]. In addition, hydrogen fluoride accumulating in the capillary tracheae is discharged into the upper tract because of ciliary action and enters the gastrointestinal system where it is ultimately absorbed by the gastrointestinal tract. This can cause gastrointestinal symptoms such as nausea, vomiting, and heartburn [14,16,17,19]. In this study, the chief complaints of outpatients in descending order were sore throat, headache, cough, and eye irritation, which are consistent with upper respiratory tract infection irritation symptoms due to hydrogen fluoride [20]. Many hospitalized patients who complained of gastrointestinal symptoms initially reported respiratory symptoms. Thus, the transition to gastrointestinal symptoms such as nausea and abdominal pain could be observed. Gastrointestinal symptoms such as nausea, vomiting, and abdominal pain are thought to be caused by bronchial ciliary action, which releases hydrogen fluoride into the gastrointestinal tract; this could have been caused or exacerbated by psychological stress. In addition, 19.1% of patients had headache as their chief complaint. Hydrogen fluoride can generate an irritating odor even at concentrations less than 1 ppm [9,21]. Furthermore, its potential to cause neurological symptoms may induce headache [19,22].

Fluoride ions are lipophilic and can easily penetrate the body, affecting the body by combining with calcium or magnesium [6,23]. Thus, hydrogen fluoride exposure can result in local symptoms such as burns as well as electrolyte abnormalities (e.g., hypocalcemia and hyperkalemia) by changing the concentrations of electrolytes such as calcium, magnesium, and potassium [20,24-28]. In this study, all outpatients had serum calcium within the normal range, and serum calcium did not vary with respect to the distance from the accident site. However, serum potassium was significantly higher in patients closer to the accident site. A study involving hydrofluoric acid burn cases reported that a delayed form of hyperkalemia can occur after exposure to hydrofluoric acid [24,29]. There are 2 possible mechanisms underlying the increase in blood potassium after fluoride exposure. The first is that fluoride ions deactivate Na+−K+ ATPase to increase extracellular potassium. The second involves the activation of the Na+−Ca2+ ion exchanger by fluoride ions, increasing intracellular calcium, which in turn activates calcium-dependent potassium channels to export potassium extracellularly. However, Vohra et al. [30] maintain that hyperkalemia is a phenomenon observed in some cases of hydrofluoric acid exposure and that the relationship between fluoride exposure and hyperkalemia remains unclear. Whether the high potassium concentrations in the patients who were close to the accident site were indeed due to hydrogen fluoride exposure will have to be identified through a detailed investigation in the future. In addition, hematocrit, and serum glucose increased significantly with decreasing distance to the accident site, even after adjusting for age and sex. Although it is possible that the increase in serum glucose in patients close to the accident site was due to physical and psychological stress, this also requires detailed study in the future. No lower respiratory tract lesions including pulmonary edema or interstitial pneumonitis were observed in any of the patients. Only 4 out of 48 subjects had abnormal pulmonary functioning test findings; however, a significant difference was not observed with respect to exposure distance. These results demonstrate that the effects of this accident were mostly limited to the upper respiratory tract. Furthermore, it can be assumed that the extent of hydrogen fluoride exposure was not to the point of being absorbed into the blood via the alveoli. However, 3 hospitalized patients had hemoptysis symptoms with findings of bronchitis caused by hydrogen fluoride. In addition, the majority of early patients visiting the emergency room complained of respiratory symptoms and were treated with calcium gluconate inhalation therapy. Thus, the patients exposed to relatively high concentrations of hydrogen fluoride in the initial phase of the accident suffered acute health effects severe enough to cause respiratory damage. Meanwhile, the patients who were victims of the secondary health damage caused by the hydrogen fluoride that subsequently spread through the community suffered acute health effects limited to the upper respiratory tract.

Hydrogen fluoride absorbed into the body has a half-life of 2–9 h in the blood [31], and approximately 60% is excreted into the urine within 24 h. Meanwhile, 99% of fluoride compounds not excreted are deposited in the bones as fluorapatite [32], which may lead to bone disease and fluorosis with long-term exposure [23,32-35]. In this study, urine fluoride measurements as a biological index of hydrogen fluoride accumulation in the body were attempted, but meaningful results could not be obtained, because the institution and equipment required for the test were unavailable during the early phase of the exposure. The mean and maximum urine fluoride levels of 66 samples analyzed after 2 weeks were 0.39 and 1.6 mg/L, respectively, both of which are below the threshold value. The threshold values of urine fluoride before and after work are reported to be 3 and 10 mg/g Cr by the ACGIH and 4 and 7 mg/g Cr by the German Research Federation (DFG). Urine fluoride can be affected by various conditions such as water intake and sweating; therefore, creatinine-adjusted values must be calculated. The creatinine-adjusted concentration is calculated by dividing the measured concentration by the urine creatinine concentration. However, this study has a limitation in that adjusted values were not calculated because urine creatinine was not measured. Since the urine creatinine concentration in a typical adult is 1.0–1.6 g/L, it can be assumed that the urine fluoride levels of the patients in this study were still below the threshold regardless of the fact that adjusted creatinine could not be calculated. Although our estimation of the initial exposure circumstances is limited by late urine sampling in outpatients, it can be assumed that the patients were not significantly exposed to hydrogen fluoride for at least 8 days after the accident.

There are few cases in the literature reporting the health effects of hydrogen fluoride spills in local communities. The most well-documented case in the literature is the hydrogen fluoride spill that occurred in Texas in 1987 [1,36]; although accidents occurred in Oklahoma in 1988 and Mexico in 1991, there were no studies systematically documenting the health effects due to these accidents [37-39]. In the accident that occurred in Texas in 1987, 24 tons of hydrogen fluoride were spilled by an oil company and 3,000 residents within an 800-m radius evacuated the area within 20 min of the accident. The acute health effects in patients visiting 2 hospitals after the accident were analyzed; the major symptoms reported by the patients included eye irritation (56%), sore throat (21%), headache (20.6%), and shortness of breath (19.4%), which are similar to the symptoms reported by the patients in the present study [1]. In addition, in a study that followed 10,811 residents 2 years after exposure, the extent of hydrogen fluoride exposure as well as respiratory and eye symptoms exhibited a significant dose–response relationship; furthermore, 30–40% of the people in the high-exposure group reported respiratory and eye symptoms even after 2 years [36]. These results may have been affected by a potential recall bias. However, the fact that a substantial proportion of subjects in a large-scale follow-up study showed symptoms even after 2 years warrants detailed follow-up regarding the long-term health effects caused by the Gumi hydrogen fluoride spill as well.

It is essential to evaluate the extent of exposure of every individual in order to precisely identify the health effects following the spill. However, it was impossible to measure direct exposure indices such as the atmospheric hydrogen fluoride concentration with respect to distance or the individual biological exposure index in the present study. Therefore, variables reflecting exposure levels, the timing of hospital visits after the accident, and the distance from the accident site at the time of accident were analyzed as indices that indirectly reflect the exposure level. Regarding the analysis of chief complaints with respect to the distance from the accident site, patients who reported shortness of breath as a chief complaint were closer, while complaints of rhinorrhea and no symptoms were more frequent with increasing distance. The results are concordant with the assumption that more cases of lower respiratory tract symptoms (e.g., shortness of breath) would be observed in patients closer to the accident site because of the exposure to higher concentrations of hydrogen fluoride. Meanwhile, the fact that patients who reported rhinorrhea as a chief complaint were far away from the site suggests that hydrogen fluoride exposure is unlikely to cause rhinorrhea. Another index measured in the present study was the timing of hospital visits. Early patients reported shortness of breath and nausea as chief complaints, while late patients reported cough and rhinorrhea. It can be assumed that the early patients, who visited within 3 days, were exposed to high concentrations of hydrogen fluoride, while the late patients, who visited after 4 days, included many patients with upper respiratory tract symptoms such as cough and rhinorrhea in addition to those who reported symptoms caused by low concentrations of hydrogen fluoride due to secondary community contamination.

One unusual aspect of this accident is that the hospital visits exhibited a bipolar distribution. The distribution of patients visiting the hospital showed 3 peaks on the first, eighth, and twelfth day from the accident; the decreases between the eighth and twelfth days are because the ninth and tenth days were weekend days. Thus, it can be assumed that the local residents postponed hospital visits until Monday. Most outpatients started visiting the hospital beginning on the sixth day after the accident, increasing until the twelfth day. Further, 9 out of 12 hospitalized patients were admitted 1 week after the accident. Diluted fluoride may take time to penetrate the tissues, and symptoms may be delayed for 2–3 days [27,40]. In addition, the possibility that the hydrogen fluoride lingered in the local community cannot be excluded. Therefore, it was possible that the patients’ symptoms could have developed after a certain delay from the accident or the mild initial symptoms could have worsened over time. However, this likelihood alone cannot explain the fact that the number of outpatients peaked 12 days after the accident and the total number kept increasing for over 16 days. The accident occurred while the season was changing when upper respiratory tract infections are common. Therefore, many residents may have believed upper respiratory tract infection symptoms such as cough, rhinorrhea , and sore throat were related to the hydrogen fluoride. As a consequence, some of the outpatients visiting because of hydrofluoric acid exposure may have, in fact, had unrelated simple upper respiratory tract infection symptoms. The inadequate responses of the government and relevant organizations to the hydrogen fluoride spill may have increased the distrust of residents, and the stimulating news reported daily may have had psychological effects. It will be necessary to consider the psychological and physical effects caused by hydrogen fluoride exposure as well as the relationships between hydrogen fluoride exposure and physical symptoms through detailed assessments of health effects in the future. Only with the assessment of psychological components, the whole effect of hydrogen fluoride exposure on health can be precisely determined [41].

In this study, the data analysis of the acute effects of hydrogen fluoride was limited because of a lack of information about the medical history and exposure level of the early patients. In particular, health effect evaluation and data collection were not performed for the firefighters or public officials on site, whose health risk was of greatest concern. For the early patients who visited the emergency room, medical history surveys were rarely performed and clinical tests could not be performed. Detailed medical history surveys and clinical tests were impossible because of the disaster triage situation. More information could have been gathered and this information could have aided patient care if the medical staff of the Departments of Occupational and Environmental Medicine and Emergency Medicine had cooperated immediately after the accident. Although some atmospheric measurements of the hydrogen fluoride concentration were taken after the spill, they could not be used as an index to clarify the exposure level, as they were neither systematic nor precise. Another limitation of the study is that a considerable amount of time had elapsed before biological samples for the biological exposure index analysis were stored and structured surveys were administered. This study included a total of 1,890 patients, accounting for 15.4% of the 12,243 people who received treatment for hydrofluoric acid reported by the Gumi Municipal Government until October 21, 2012. Excluding the 5,261 patients who visited the temporary on-site free clinic, the present cohort accounts for 27.0% of 6,982 patients. The facts that not all of the patients were surveyed and data from only one hospital were analyzed are also limiting factors of this study. This incident warrants the establishment of a system that would enable the medical staff of the Department of Occupational and Environmental Medicine to immediately intervene in situations involving environmental disease due to chemical substances. The outpatient clinic of the Department of Occupational and Environmental Medicine also needs to develop a system to identify the level of exposure of individual patients and be equipped to face large-scale environmental disasters that occur without warning.

The chronic health effects of fluoride and hydrogen fluoride, including skeletal and dental fluorosis, have only been reported among residents living in areas where drinking water contains high concentrations of fluoride or workers who have long-term exposure to fluoride during the aluminum smelting process [9,32,42,43]. This accident was a one-time acute spill. On the basis of the symptoms and clinical test results from outpatients, neither the fluoride exposure level nor the exposure period is believed to have been sufficient to cause skeletal or dental fluorosis. However, monitoring the health of exposed residents is necessary because of the unique aspects of the Gumi spill and the lack of data about the long-term health effects after acute hydrogen fluoride exposure. Considering that after the spill in Texas in 1987, several cases in the high-exposure group had symptoms persisting even after 2 years, the possibility of prolonged physical and psychological symptoms in local community members cannot be ruled out. In particular, the initial investigation was inadequate regarding mental health aspects such as depression and post-traumatic stress disorder. Therefore, continuous follow-up observations on long-term psychological and physical health are necessary.

## Conclusion

The patients exposed to relatively high concentrations of hydrogen fluoride in the initial phase of the accident suffered acute health effects severe enough to cause respiratory damage. Meanwhile, the patients who were victims of the secondary health damage caused by community spread suffered by symptoms limited to the upper respiratory tract such as sore throat, headache, cough, and eye irritation. However, monitoring the health of exposed residents is necessary because of the unique aspects of the Gumi spill and the lack of data about the long-term health effects after acute hydrogen fluoride exposure.

## Competing interests

The authors declare that they have no competing interests.

## Authors’ contributions

All authors had access to the data and played a role in writing this manuscript. JSK conceived and designed the study. SYY and SYC were involved in writing the manuscript. SIU and JAK performed the data collection. JSK and JYN performed the statistical analysis, the interpretation of data. KHW had critically revised the manuscript. All authors read and approved the final manuscript.
